# The path towards consensus genome classification of diffuse large B-cell lymphoma for use in clinical practice

**DOI:** 10.3389/fonc.2022.970063

**Published:** 2022-10-27

**Authors:** Matias Mendeville, Margaretha G. M. Roemer, G. Tjitske Los-de Vries, Martine E. D. Chamuleau, Daphne de Jong, Bauke Ylstra

**Affiliations:** ^1^ 1Department of Pathology, Cancer Center Amsterdam, Amsterdam UMC location Vrije Universiteit, Amsterdam, Netherlands; ^2^ Department of Haematology, Cancer Center Amsterdam, Amsterdam UMC location Vrije Universiteit, Amsterdam, Netherlands

**Keywords:** diffuse large B-cell lymphoma (DLBCL), next generation sequencing (NGS), consensus classification, genomics, bioinformatics

## Abstract

Diffuse large B-cell lymphoma (DLBCL) is a widely heterogeneous disease in presentation, treatment response and outcome that results from a broad biological heterogeneity. Various stratification approaches have been proposed over time but failed to sufficiently capture the heterogeneous biology and behavior of the disease in a clinically relevant manner. The most recent DNA-based genomic subtyping studies are a major step forward by offering a level of refinement that could serve as a basis for exploration of personalized and targeted treatment for the years to come. To enable consistent trial designs and allow meaningful comparisons between studies, harmonization of the currently available knowledge into a single genomic classification widely applicable in daily practice is pivotal. In this review, we investigate potential avenues for harmonization of the presently available genomic subtypes of DLBCL inspired by consensus molecular classifications achieved for other malignancies. Finally, suggestions for laboratory techniques and infrastructure required for successful clinical implementation are described.

## Introduction

Molecular diagnostics of cancer has entered a new era, propelled by advances in omics- and bioinformatic technologies that provide a new layer of characteristics for tumor classification. In general, current state-of-the-art diagnostic pathology categorizes tumors using phenotypic macro- and microscopic and immunohistochemical (IHC) characteristics, combined with molecular assays for single or limited numbers of markers like PCR, and fluorescent *in situ* hybridization (FISH). Analyses of highly complex omics data by bioinformatic technologies have identified molecular patterns and pathways that underly biologically distinct, and thereby newly recognized categories. *Vice versa*, accepted diagnostics distinct categories may proof to be molecularly so closely related they may even be combined into a single entity.

Diffuse large B-cell lymphoma (DLBCL), the most prevalent type of non-Hodgkin lymphoma and the focus of this review, is characterized by a complex, heterogeneous tumor biology that is reflected in clinical heterogeneity (1). This is evident from a wide outcome spectrum with cure for 60% of patients treated with standard immune-chemotherapy (R-CHOP) and disease progression for the other 40% of which the far majority eventually succumbs due to relapsing and/or refractory disease (2, 3). Since 2000, omics information started to contribute layers of comprehensive biological information to the diagnosis of DLBCL (4). At that time, RNA expression profiling by means of microarray analysis followed by unsupervised clustering revealed a relatively simple dichotomous distinction based on cell-of-origin (COO) (5). For universal application in daily clinical practice, this distinction was translated into various algorithms that relied on classic immunohistochemistry (IHC) assay data rather than complex RNA analytics. This undoubtedly aided to have DLBCL COO classification to be included in the updated 4^th^ edition of the World Health Organization (WHO) Classification for Hematolymphoid Malignancies in 2016 (6). Nonetheless, it was never widely applied outside clinical trials, largely since the clinical implications ultimately proved to be limited (7–9). Almost 20 years after the RNA-based COO classification concept, several independent studies proposed DNA-based subtyping by next-generation sequencing (NGS) as an alternative means to capture the biological heterogeneity of DLBCL and to supersede or complement COO classification (10–13). The different DNA-subtyping studies bear significant similarities, but also differ in some *a priori* concepts, applied technologies, bioinformatical approaches and ultimately in part in recognized genomic subtypes (14, 15). These differences preclude uniform classification, which is a quintessential step towards clinical implementation and essential to perform meaningful clinical trials (16–18).

## Molecular classifications of DLBCL

### Classifications based on RNA-expression

The more than 20-year-old RNA-based COO classification recognizes 2 major molecularly distinct classes considered to reflect different stages of B-cell differentiation; activated B-cell (ABC) and germinal center B-cell (GCB) while a small group of patients remains ‘unclassified’. Both in the primary discovery studies and various subsequent validation studies, patients with a GCB-type DLBCL consistently showed a better prognosis under guideline therapy than patients with an ABC-type DLBCL (4). The differential clinical outcomes coupled with distinctive underlying biology served as a justification for differential treatment. In the years that followed it became clear however that the complex and heterogeneous biology of DLBCL was not fully captured by this simple dichotomous classification (5). In particular, phase 2 and phase 3 clinical trials that either used COO as an inclusion parameter, or were *post-hoc* analyzed based on COO class, failed to demonstrate differential improvement of outcome for patients receiving experimental, targeted treatment alternatives (7, 8, 19).

This does however not imply that RNA-based information would not provide essential information to dissect DLBCL biology, as specific host-immune response signatures could already be identified in the early 2000s (20). Most recently, deconvolution algorithms using known cell type specific RNA signatures to computationally infer cellular components from bulk RNA data have allowed to further dissect information on tumor features as well as non-malignant tumor immune microenvironment (TME) features. Thereby, the original GCB class was further divided into three to four differentiation phases (germinal center, dark zone, precursor memory B-cell, light zone) and ABC into two phases (pre-plasmablast, plasmablast/plasmacell). Hence, TME analysis from RNA expression data provided complementary signatures that could further and largely independently describe DLBCL biology in a clinically meaningful manner (21).

### DLBCL defining DNA-alterations and subtyping approaches

The first larger DNA-based next-generation sequencing (NGS) studies for DLBCL that were undertaken revealed a spectrum of mutations, numerical chromosomal copy number aberrations (CNAs) and translocations that were largely characteristic for either of the RNA expression-based COO classes (22–27). For example, mutations in the chromatin modifying genes *CREBBP, KMT2D* and *EZH2*, were described as characteristic of GCB-type DLBCL and chromosome *18q* gain and *MYD88* mutations characteristic of ABC-type DLBCL. Apart from these few COO-characteristic DNA alterations, the majority was shown to be only limitedly overrepresented in either class, explanatory for the extensive genetic heterogeneity of DLBCL.

**Figure 1:**
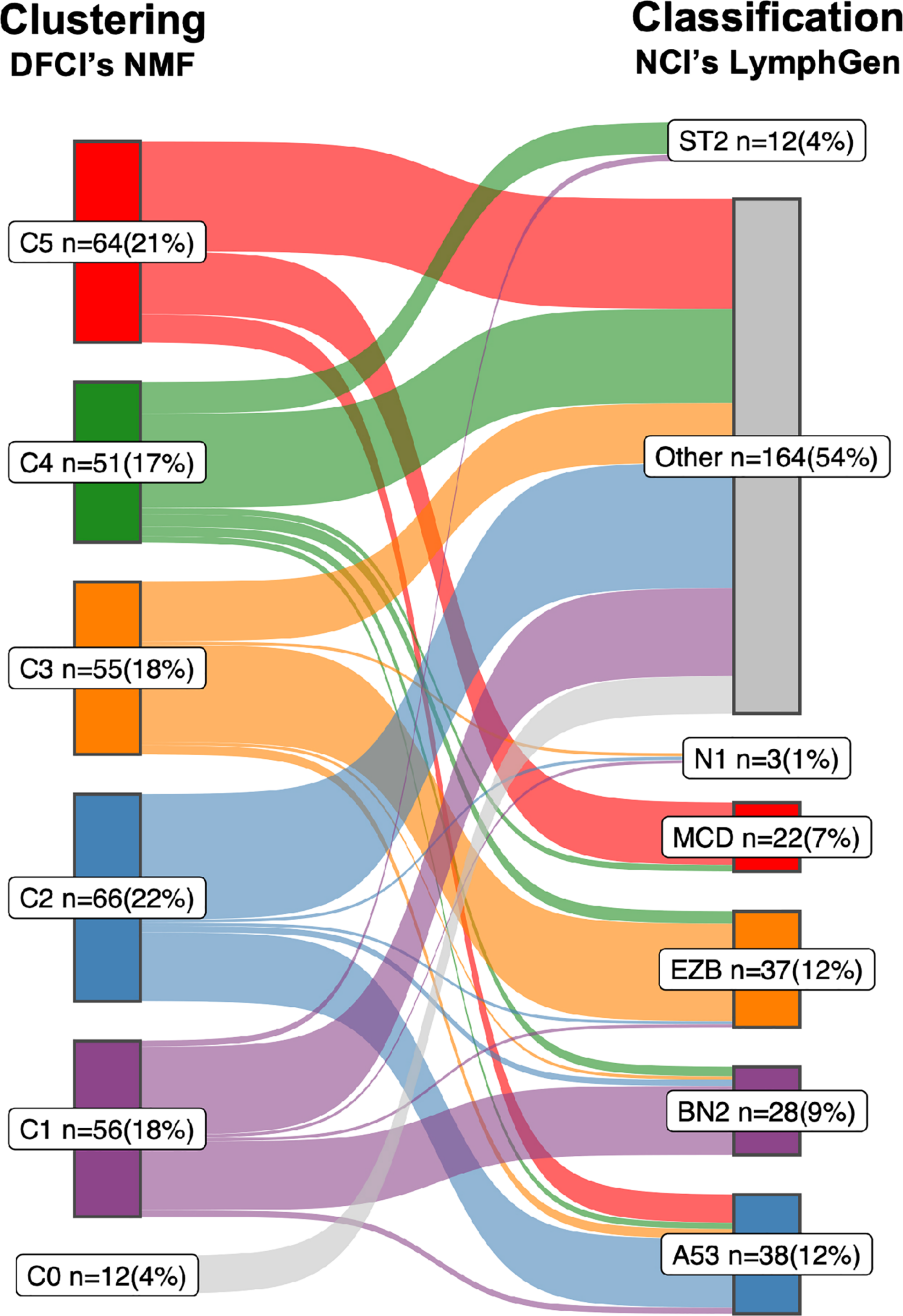
Sankey diagram comparing the two DLBCL subtypes. A Sankey diagram was constructed to illustrate how the LymphGen (12) and NMF (10) subtyping systems compare, as described in **Box 1**. Therefore the NGS data of 304 diffuse large B-cell lymphoma (DLBCL) cases published by the Dana Farber Cancer Institute (DFCI) (10) was used as input. Left stage: Clustering by means of non-negative matrix factorization (NMF). Right stage: Classification by means of LymphGen algorithm. Flows between the subtypes resemble DLBCL cases and are labelled according to their molecular counterpart; C1/BN2, purple; C2/A53, blue; C3/EZB, orange; C4/ST2, green; C5/MCD, red; samples not assigned to a cluster (NMF, C0) or unclassified (LymphGen, Other) are in gray. Each subtype with numbers of samples (n) and percentage of total (=304).

In 2018, research groups from the National Cancer Institute (NCI) and the Dana Farber Cancer Institute (DFCI) independently and practically simultaneously proposed DNA-based subtyping approaches based on whole exome sequencing (WES) (1, 10, 11). The NCI group made a first step towards harmonization of the two approaches by, like DFCI, also including CNAs to their classification which resulted in the LymphGen algorithm (12). The DFCI- and NCI studies included retrospectively collected patient cohorts and identified 5- and 7 genomic subtypes, respectively. Encouraging is that despite the different cohorts and bioinformatical approaches, both defining features and the resulting subtypes are largely overlapping (**Figure 1** and **Box 1**). Other groups, with other cohorts using overlapping bioinformatical approaches have been able to reproduce these subtypes by and large (13, 31–33), including unpublished results by the authors of this review. This all provides confidence that a DNA-based characterization of DLBCL has the potential to disentangle the biological heterogeneity that underlies DLBCL’s clinical heterogeneity.

Box 1 **Comparison of the LymphGen and NMF subtyping systems.**

The correspondence between the NCI’s LymphGen and DFCI’s NMF subtypes is 75% based on the 63.1% of patients classified by the LymphGen algorithm (12). If also the LymphGen unclassified samples are considered, the overall agreement between the two subtyping systems is around 50%. Approaches: Both studies performed comprehensive genomic profiling to detect somatic mutations, CNAs and translocations. Because of the lack of matched normal tissue for most samples, both studies applied custom computational pre-processing techniques to eliminate sequencing artifacts and distinguish somatic and germline variations. The DFCI group performed WES on a series of tissue biopsies of 304 patients with primary DLBCL. Samples were from 4 different trials and cohorts, of which 55% were derived from FFPE tissue, and 44% had matched-normal tissue availability (10). The NCI group performed WES on a series of fresh-frozen DLBCL tissue biopsies of 574 patients for which 96.5% were primary DLBCL tissues and the other 3.5% from relapsed or refractory, without matched-normal tissue (12). Below is a short summary of the most defining features which the NCI and DFCI proposed subtypes have in common. For a more comprehensive overview on details of their differences and commonalities we refer to a recent review by Crombie et al. (28).

i. The C1 subtype recognized by DFCIs NMF algorithm finds its analogue in the BN2 subtype recognized by NCIs LymphGen algorithm. Combined, the two algorithms determined 21 defining genetic alterations, of which eight overlap. Overlapping genes include *BCL6* translocations, alterations in *NOTCH2* signaling genes and mutations targeting the NF-kB pathway. Furthermore, the C1/BN2 subtype is enriched for, but not restricted to ABC-type, and shows a favorable outcome. The C1/BN2 alterations form a genetic basis of immune evasion corresponding to mutations seen in marginal zone lymphoma. Non-overlapping genes include mutations of *B2M*, *FAS*, *HLA-B* and translocations of *PD-1* ligands.
ii. The NMF-C2 subtype is analogues to the LymphGen-A53 subtype. Both have characteristic *TP53* inactivation, and a high degree of genome instability as reflected by the prominence of genome-wide CNAs. This subtype is not significantly enriched for either of the two COO types, which underpins that the original COO dichotomy was indeed an oversimplification of DLBCL biology. Overall survival of this C2/A53 subtype under R-CHOP treatment is unfavorable. A notable difference between the two subtypes is the high number of discordant subtype-defining features (36 from 41), including driver alterations such as chromosomal deletion of the *CDKN2A* locus (9p.21).
iii. The NMF-C3 subtype is analogues to the LymphGen-EZB subtype, with a relatively high concordance of subtype-defining alterations (10 out of 18); including translocations of *BCL2*, and mutations in chromatin modifying genes. Discordant features include amplification of the *REL* locus (2p16.1) and mutations of *FAS.* The C3/EZB subtype represents classic GCB-type DLBCLs, and the genetic features are to a large extent alike follicular lymphoma (FL), which suggest that these DLBCLs represent transformed FL (29). Clinically, C3/EZB subtype tumors are considered of most high risk within the GCB-type of DLBCLs. Notably, also the RNA-based DHITSig is enriched in this subtype and used to further subdivide EZB.
iv. The NMF-C4 subtype is analogous to the LymphGen-ST2 subtype. C4/ST2 subtype defining alterations affect BCR/PI3K signaling, the JAK/STAT pathway, and histone genes. Most of these DLBCLs belong to the GCB-type with favorable outcome. Few alterations linked to this subtype are concordant between the two classification systems (6 out of 24). The less defined nature of this subtype is further underpinned by a recent study suggesting that this subtype may be further subdivided into two subtypes with divergent biology: a *TET2/SGK1* and a *SOCS1/SGK1* subtype (13).
v. The NMF-C5 subtype is analogues to the LymphGen-MCD subtype. Nine of the 24 characteristic alterations overlap which include mutations in genes associated with extranodal involvement (*MYD88*, *CD79B, TBL1XR1*). This C5/MCD subtype is highly enriched for ABC-type DLBCLs and is the subtype with the least favorable survival under R-CHOP treatment. Discordant alterations include other markers of immune evasion (mutations of *HLA-B* and translocations of *PD-1* ligands) and copy number gains of chromosomal arms 3q and 18q.
vi. Finally, the LymphGen classification describes the N1 subtype which is characterized by *NOTCH1* mutations. This subtype occurs in less than 2% of DLBCLs **Figure 1** and has the worst survival among the LymphGen subtypes. This subtype is not recognized by the NMF algorithm with the DFCI cohort. Also, when we extend the DFCI cohort with another 500 DLBCLs treated with R-CHOP, the NMF algorithm still does not recognize this class (authors unpublished results).

### The bioinformatic approaches of the current DNA-based subtyping systems for DLBCL

The DFCI group used unsupervised clustering combined with alteration-centric features (**Box 2**). Driver alterations were discriminated from passengers, thereby reducing the genetic dataset to 158 features. Non-negative matrix factorization (NMF), an unsupervised clustering algorithm that detects patterns of co-occurring features and assigns a subtype to each included tumor, was used. The number of clusters to be identified was predefined between 4 and 10, which is actually an arbitrary choice. The NMF algorithm identified the optimal stability of clusters to be represented by 5 DLBCL groups of similar sizes, which the authors labelled as C1 to C5.

Box 2 **Genome feature definition and subtyping algorithms.**

The two proposed DNA-based subtyping systems differ in their bioinformatic approaches for i) genomic feature definition, and ii) subtype identification (10, 12):

i. To define genomic features a gene-centric approach can be applied that combines all DNA alterations that impact the same gene into 1 feature, independent of whether they are a mutation, translocation or CNA. For example, a point-mutation of *CDKN2A* and a deletion of the *CDKN2A*-locus 9p.21 would be recognized as 1 feature. Alternatively, an alteration-centric approach regards each DNA alteration type separately, independent of their location in the genome. In the example of *CDKN2A*, the mutation and 9p.21 deletion are regarded as two separate features.
ii. Also machine learning algorithms for patient subtyping can generally be divided in 2 main approaches, supervised or unsupervised (30). The supervised approach uses predefined classes to construct a classification rule from the features. An unsupervised approach leaves it to the algorithm to identify a number of subtypes that are composed of feature characteristics prioritized by the algorithm. Semi-supervised learning would be where some prior knowledge on classes and or features is given.

The NCI group used semi-supervised clustering combined with gene-centric features (**Box 2**). The prior knowledge given were four predefined classes, each composed of 1 or 2 specific DNA "seed" alterations: MCD (seed is co-mutation of *CD79B* and *MYD88* ^L265P^), BN2 (seed is *NOTCH2* mutation or *BCL6* translocation), N1 (seed is *NOTCH1* mutation) and EZB (seed is *EZH2* mutation or *BCL2* translocation). Finally, the algorithm selected the additional genomic features that had the strongest association with the four classes through an iterative approach. All patient samples were included for classification with this 4-class algorithm, yet of the entire cohort, only 46% of cases could be assigned (11). In the remaining 54% of cases in the NCI cohort recurrent alterations of *TP53* (25%), *TET2* (10%) and *SGK1* (6.9%) were identified. This prompted the NCI group to refine and extend the four classes with two additional classes: A53 (seed is mutation and/or CNA of *TP53*) and ST2 (seed is mutations of *SGK1* and *TET2*), resulting in six seed classes (12). Subsequently, a Bayesian predictor model titled “LymphGen” was developed, which calculates for each individual tumor the subtype probabilities for each of the six classes based on its genetic alterations. Tumors designated as "core" tumors were defined as being attributed to one class with a probability score of >90%. Consequently, the Bayesian predictor allows tumors to be assigned to multiple classes. Those with a probability score greater than 90% for more than one class are the so called “genetically composite” tumors. Tumors with a probability score of 50%-90% for one single class were termed “extended” class members. Tumors with few subtype-specific genetic alterations were left unclassified. Thereby, the then 6-class LymphGen algorithm assigned 63.1% of cases of the NCI cohort (12). Later, the RNA expression-based *MYC* double-hit signature (DHITSig), previously developed by others (34), was added as a surrogate for *MYC* translocation status to split the EZB class in *MYC* positive and *MYC* negative cases.

### Critical evaluation of the current subtyping approaches for DLBCL

Despite the different choices in feature identification and machine learning algorithms (**Box 2**), the NCI and DFCI groups recognize a similar and extensive underlying biological heterogeneity of DLBCL. Some subtypes are already more similar than others. For example LymphGens MCD/NMF C5, LymphGen A53/NMF C2 and LymphGen EZB/NMF C3 are already relatively consistently defined. An important difference is that the LymphGen algorithm does only assign 63.1% of patients to any of their predefined subtypes, whereas the DFCIs NMF algorithm defines a number of subtypes to which 100% of the samples in the cohort are assigned. The N1 subtype is the rarest subtype and is only recognized by the NCI with the *NOTCH1* mutation seed given to the LymphGen algorithm (**Box 1**).

A small fraction of DLBCL patients (<2%) carry *NOTCH1* mutations which infers potential specific sensitivity to Ibrutinib, a Bruton’s tyrosine kinase (BTK) inhibitor. Due to its low frequency, the N1 subtype is not recognized using unsupervised techniques in relatively small series. The size of the currently studied cohorts has been too small, hence underpowered, to detect such rare genomic subtypes by unsupervised analysis. Unknown small genomic subtypes can only get recognized once the sample size is sufficiently large, as exemplified by Curtis et al. for breast cancer (35). Rare subtypes like N1 may be characterized by very specific biological characteristics that make them uniquely targetable with specific potent inhibitors and thereby highly relevant to be recognized. As an example from another cancer entity, in about 1% of metastatic colorectal cancers the *ERBB2* oncogene on chromosome *17q* is amplified, which can be effectively targeted by trastuzumab and neratinib and results in high response rates in these tumors (36–38). Likewise, 4-5% of non-small-cell lung cancers have a translocation of the *ALK* gene, which can be effectively targeted by the ALK inhibitor crizotinib (39).

Not recognized by either NCI or DFCI are the actual high-grade B-cell lymphoma (HGBCL), B-cell lymphomas with *MYC* translocation together with either *BCL2* and/or *BCL6* translocation (double hit/triple hit). Unsupervised NMF clustering theoretically might be able to recognize this group as a subtype but, like the N1 subtype, it may have remained undetected as a result of the limited number of *MYC*-translocation positive DLBCLs in the DFCI dataset. The DHITSig signature used by the NCI is a surrogate marker to recognize a MYC subtype and troublesome for various reasons. First it is not DNA alteration derived and requires a different assay, namely RNA expression analysis. Second, the name of this signature is deceiving since it implies a genetic context of HGBCL, whereas only 64% of DHITSig-positive GCB-type DLBCLs actually carry a *MYC* translocation and 52% are actual double hit/triple hit DLBCLs (34). Third, also other lymphoma classes besides *HGBCL* double hit/triple hit such as Burkitt lymphoma score positive for DHITSig. This RNA DHIT signature is thus not specific for either *MYC* translocation or HGBCL (40, 41).

Besides the choice of subtyping algorithms, the NCI gene-centric versus DFCI alteration-centric choices for genetic features deserve attention (**Box 2**). The easiest solved are the focal chromosomal CNAs, aberrations smaller than 3Mb (42) which only encompass one or few genes, and can therefore be combined in a gene-centric fashion (43). The choice between alteration- or gene-centric is not obvious for the larger-scale chromosomal CNAs since they harbor hundreds of genes. Rather than rationalizing a choice between a gene-or alteration-centric approach, the machine learning algorithms can be offered data processed in either manner and side-by-side evaluated for best subtyping performance.

Although the unsupervised clustering choice is an elegant data-driven approach to identify subtypes (17, 44, 45), in the end a classifier, like LymphGen, will need to be built to diagnose individual patients in daily clinical practice, which dictates another step towards harmonization.

## Towards a unifying classification for DLBCL; Lessons learned from other tumor types

### Two steps towards clinical implementation of a DNA-based classification of DLBCL

The currently proposed DNA-based subtypes will be the basis for a unified biological classification that may require a two-step strategy (28). Step 1 would involve harmonization of the current DNA-subtyping systems into a single unified classification, Step 2 would be the development of a reproducible and widely applicable molecular diagnostic assay; certified, as well as cost- and time-effective to enable clinical implementation. This exposes various challenges, from the choice of laboratory technique, subtype-defining DNA alteration features and interpretation to classification algorithms and bioinformatic procedures.

### A universally accepted classification is a prerequisite to improve patient management

Harmonization into a single classification is a first requirement for implementation in diagnostic routine. Objective, reproducible, and conclusive subtype definition for each patient sample, combined with a detailed understanding of the tumor biology of each defined DNA-class, will enable to explore clinical consequences of such classification, preferably in clinical trials (46). For various organ-specific malignancies molecular classifications for tumor families have now been standardized and integrated in the 5^th^ edition series of the WHO Classifications and are starting to be implemented in the diagnostic workflow for those settings that have access to the technology (47–49). The road towards this level of applicability has been achieved with several research groups proposing their individual molecular classification as a starting point, at different moments in time and with different laboratory and bioinformatical techniques, as is exemplified by the classification of breast cancer, central nervous system (CNS) tumors and colorectal cancer (48, 49).

### Lessons learned from classifications that are universally agreed upon for other solid malignancies

Probably breast cancer classification is one of the most successful early examples. An RNA-based classification for breast cancer found its way already into the 4th edition of the WHO Classifications of Breast Tumours in 2012, which was further expanded upon in 5th edition (47). it recognizes 5 molecular classes; each with different prognosis but also different treatment recommendations. The existing close transatlantic collaborations undoubtedly facilitated consensus formation, characterized as “organic” allowing different biological and bioinformatical perspectives to converge (46, 50, 51). Once a consensus classification was established and reproducible assays were developed, exploration of personalized and targeted treatment approaches could be effectively explored to identify bespoke treatment modalities, amongst others in the multi-armed I-SPY clinical trials (52).

From the point of view of development of a molecular-based consensus classification, the present WHO classification for CNS tumors is an impressive result of intensive collaboration leading to a highly refined molecular classification. In 2014 a group of neuro-oncological pathologists, physically converged in 2014 in Haarlem (NLD) and prepared a clinically relevant histo-molecular diagnostic consensus classification, whilst reducing interobserver variability (53), which soon was implemented in the 4th edition of the WHO Classification of CNS Tumors (54). Subsequently, a largely novel approach was taken by means of genome-wide DNA methylation analysis where the large spectrum of CNS tumors were recognized by methylation profiles combined with a form of dimension reduction called t-distributed stochastic neighbor embedding (t-SNE) (55). The t-SNE methylation test alone allows for diagnoses of the large majority of CNS tumors, not seldomly more detailed and/or reliable compared to the histo-molecular diagnosis, resulting in redefinition of these entities. The collaborative effort with inclusion of samples and intellectual input from many research groups across the world as well as extensive discussions in the Consortium to Inform Molecular and Practical Approaches to CNS Tumor Taxonomy (cIMPACT-NOW) (56) has helped a broad acceptance and indeed this molecular classification is now also included in the 5th edition of the WHO Classification of Central Nervous System Tumours (48, 48).

To harmonize colorectal cancer (CRC) classification, the Colorectal Cancer Subtyping Consortium (CRCSC) was formed to integrate six independently published RNA-based classifications (49). As opposed to the CNS assembly consensus, a predefined mathematical harmonization path was taken with the aim to resolve inconsistencies between the various CRC classification systems. This approach culminated in four consensus molecular subtypes (CMSs) (49) to which each CRC sample adheres to a higher (core samples) or lesser (non-core) extend. Since the context in CRC classification is so very similar to the current status in DLBCL, we here provide a summary of this CMS approach where three generic methodological steps were involved (**Box 3**).

Box 3 **Summary of the data-driven bioinformatic path to the four consensus molecular subtypes for colorectal cancer.**

Three generic methodological steps are involved in the path taken for consensus classification of colorectal cancer.

i. Independent expert team subtyping prediction on normalized raw data sets: Eighteen RNA-based CRC gene expression data sets, derived from different continents and research groups were assembled from public resources (Gene Expression Omnibus and The Cancer Genome Atlas). The data sets were compiled from various genome-wide expression analysis techniques (arrays and RNA-sequencing), different sample types (formalin-fixed paraffin embedded and fresh-frozen tissue materials) and different study designs (retrospective and prospective series, including clinical trials). The first bioinformatics step concerned central pre-processing and normalization aimed to obtain expression profiles for each of the patients of the 18 gene-expression datasets, independent of cohort or technique. Next, each of the six initial participating research teams applied their original classification algorithm to each of the 18 data sets. Thus, resulting in six classifications, with a total of 27 different subtypes for all 3,962 patients.
ii. Network analysis for consensus subtype identification: Using the six classification systems of the 3,962 patients, a network-based approach was applied to study the association between all the 27 subtypes. To detect robust clusters of recurrent molecular subtypes, an unsupervised Markov clustering approach was performed, resulting in the identification of four consensus molecular subtypes (CMSs). Of the 3962 samples, 3104 (78%) were identified as highly representative of a particular subtype and labelled as core consensus samples and the remaining n=858 as non-consensus samples. The core consensus samples were used to train the novel CMS classifier in the subsequent step.
iii. CMS classifier construction and application: To allow classifications of individual cases, which is mandatory for diagnostic routine, a classification algorithm is required. Since the data sets were created using different RNA gene expression profiling techniques across the different studies, not all genes were included in all data sets. The CRCSC first converted all 18 separate data sets into a single data set. The genes that were commonly profiled by all separate data sets were selected to allow aggregation of all 18 data sets into a single data matrix. To construct the CMS classifier, the single data matrix, CMS classes and consensus sample set were used. The consensus samples were randomly split using two-third as training and one-third as validation set, and a random forest classifier was generated to calculate a prediction value for subtype assignment for each sample, by means of bootstrapping with 500 iterations. Application of the CMS classifier on the validation set demonstrated an overall accuracy of 90%. The CMS classifier was robust enough to allow assignment of 40% of the non-consensus samples, while the rest showed heterogeneous patterns of CMS subtypes and contained biological information of more than one class.

The process to come to a single, harmonized molecular classification for DLBCL may likely be the one taken for the development of colorectal cancer CMS. For DLBCL also, a similar issue in the underlying biology result in single class (core) tumors, unclassified samples and genetically composite tumors (12, 57). What should alleviate the consensus process is that for DLBCL two, rather than the six for colorectal cancer, existing DNA-classifications as a starting point while still various independent published and unpublished (authors of this review) datasets are available.

## From DLBCL genome classification to clinical implementation

### DNA alterations required for DLBCL genome classification

**Figure 2:**
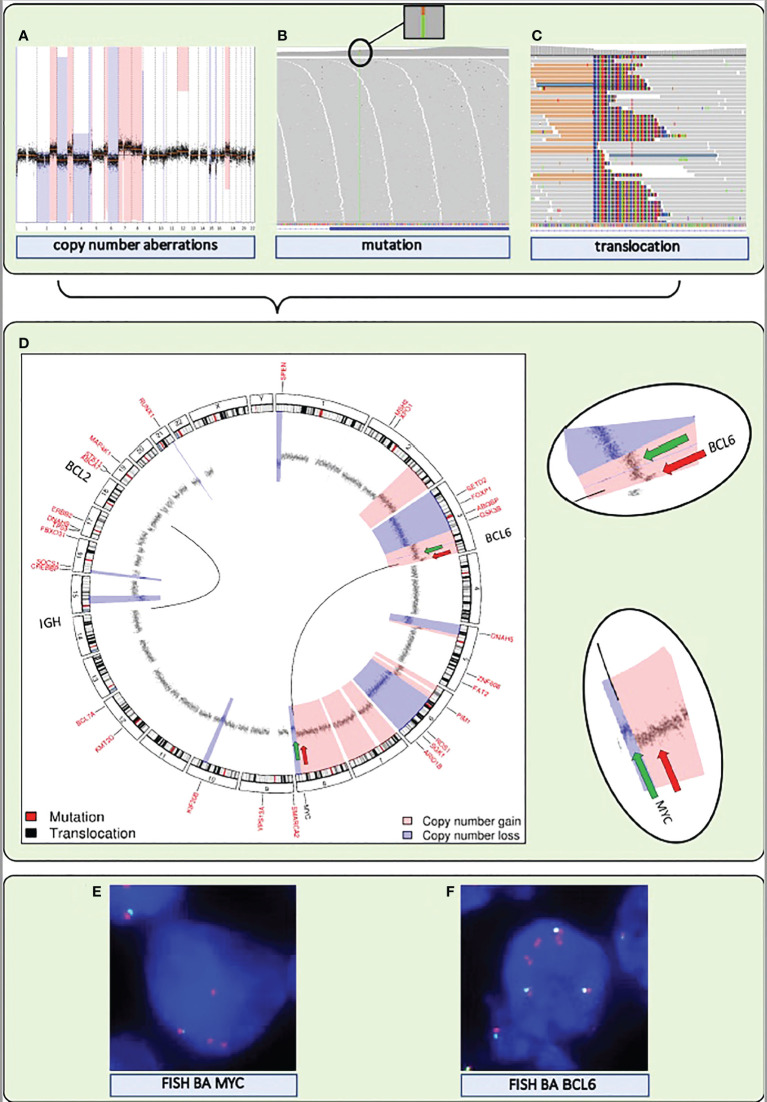
A single NGS assay to detect somatic mutations and structural variations, including translocations and CNAs, from DNA extracted from FFPE tissue. **(A–C)** Visualization of the detection by NGS of CNAs, mutations, and translocations for a DLBCL sample. **(A)** Genome-wide chromosomal CNAs. x-axis shows chromosomes 1 to 22, from left to right, y-axis shows copy number gains (light red) and copy number losses (blue). **(B)** Screenshot of high coverage (200X) NGS sequence reads aligned to the reference genome highlighting a somatic mutation in *KMT2D*. **(C)** Screenshot of high coverage (200X) NGS sequence reads aligned to the reference genome highlighting a translocation breakpoint in *MYC*. **(D)** A circular representation of the genome depicting mutations (genes denoted in small red letters), translocations (genes denoted with large black letters connected by black lines) and CNAs (inner circle: black dots are measurement bins and called losses are colored in blue and gains in light red). Green and red arrows point to the position of break apart (BA) FISH probes that were used as a control for the translocations detected by NGS. **(E)** FISH BA MYC. **(F)** FISH BA BCL6. Integrated NGS analysis explains aberrant FISH pattern: a loss (green arrow) and a gain (red arrow) at the *MYC* locus coincide with a single (green arrow) and double gain (red arrow) at the translocation partner *BCL6* locus.

Any consensus classification for DLBCL will include a combination of mutations and structural chromosomal variations (CNAs and translocations) (**Box 1**). Therefore, inclusion of this information into a single genome subtyping assay would be highly attractive. Various common laboratory and bioinformatics applications are available for mutation and CNA detection by NGS. Also NGS-based translocation detection is starting to become a cost-effective alternative for routinely used Fluorescent *in situ* hybridization (FISH) to determine translocations. (**Figure 2**). FISH benefits from a choice of worldwide commercially available probes and assays but is labor-intensive with a certain level of technical variability and subjectivity in interpretation. Thereby, NGS outperforms FISH in several ways: it avoids interobserver variability, it can be performed with small and histologically compromised materials, and it is able to identify exact translocation breakpoints on nucleotide level. An additional advantage of some of the NGS approaches is that unknown translocation partners may be identified, that may be of clinical relevance for the biological and clinical interpretation of DLBCL patients with a *MYC* translocation (40). Various combinations of NGS and bioinformatics platforms have been successfully developed in this direction (58–61).

### Assays for clinical implementation

World-wide clinical implementation of any diagnostic routine requires relatively simple assays that are applicable to routine diagnostic tissue material, such as formalin-fixed paraffin embedded (FFPE) specimens. The elaborate laboratory- and informatics infrastructure needed for current NGS or array analysis may only be available in selected settings of large medical centers or commercial providers as exemplified for CNS tumors. Favorable aspects of commercial involvement are the wide availability, extensive standardization, quality control and rapid turnover time due to high case volumes. Downsides are amongst others worldwide availability, financial dependency and commercial goals, market dominance of individual commercial providers, lack of technical transparency and development, lack of flexibility to include most recent research developments and generally lack of integrated interpretation with other pathology parameters. Another option to bring a genome subtyping assay to implementation in daily practice is to “reduce” complex molecular information to simpler and widely applicable techniques. The DLBCL-COO classification alternative is a good example; genome-wide molecular classification with elaborate bioinformatics was translated into several simple immunohistochemistry (IHC) markers, of which the Hans classification is most widely used (62). All IHC-based COO assays show limited concordance with the gold standard of RNA expression-based assays (63). This prompted the development of a digital gene expression assay based on 20 key genes that can be applied on FFPE material (64). This Lymph2Cx assay, restricted to equipment from the company Nanostring (Seattle, USA), showed high concordance with the original RNA expression-based COO classification with a 2% error rate in COO assignment (65). These characteristics, together with a short turnaround time of less than 36 hours, allowed for rapid molecular characterization of patients, making this assay a suitable middle-ground alternative for employment in research and clinical trials (19). Similar assays have been commercialized by others (66). In view of the expected high-dimensional nature of a consensus molecular classifier for DLBCL, simple translation to an IHC is not likely. Current NGS techniques are already reliably applicable for FFPE biopsy samples offered by commercial providers. It may be expected that these companies will readily offer products for consensus molecular DLBCL classification once this would be developed.

### Assay and turnaround time

A single genome subtyping assay that detects CNAs, mutations, and translocations in parallel would conceivably be most efficient in terms of labor, cost and tissue material. But is this also efficient in terms of turnaround time? A recent study showed that real-time molecular profiling of RNA-based COO determination of DLBCL is realistic to stratify patients in a timely manner, with a median turnaround time of 8 days (8). This would be a desirable timeframe for DNA-based DLBCL classification, such that based on tumor vulnerabilities, patients can be diverted after 1 or 2 cycles standard R-CHOP treatment, which is a successful approach facilitating rapid trial inclusion (67). A recent feasibility study in the Netherlands, which involves a WGS specialized non-profit organization, was performed to evaluate implementation of WGS into routine diagnostics (68). Meanwhile, they were able to optimize the turnaround time from biopsy to DNA report to 7 working days, demonstrating the potential of clinical implementation of NGS methods for these purposes.

## Application in daily clinical practice and promising future developments

### Bespoke treatment of DLBCL patients

Once validated, uniform and widely applicable, consensus molecular subtypes of DLBCL will be a sound basis to explore more effective, targeted treatment methods (1). The potential of DNA-based classification for precision medicine of DLBCL has been demonstrated in a recent retrospective analysis of a randomized phase-III trial (69). In this study, patients under 60 with two specific DNA subtypes (LymphGen’s MCD and N1) that received R-CHOP with Ibrutinib had significantly better survival (both subtypes 100% 3-year event-free survival) than patients that received R-CHOP alone (42.9% and 50%, respectively), clearly indicating the potential predictive value of the novel genomic subtypes. Next, prospective clinical trials may further explore associations with genomic subtypes and associations with targeted compounds, such as NFkB-inhibitors, PI3K inhibitors, P53-modulators and apoptosis modulators, as well as immunotherapy such as immune checkpoint inhibitors and CAR-T cell therapy. For this purpose, various dedicated next-generation designs are now proposed (70).

It is obvious to further investigate to what extent the integration of the current DNA-based and RNA/microenvironmental-based subtyping methods for DLBCL would be of added value. Adding a layer of epigenetic information as for CNS (55) or even germline genetic characteristics might be considered (71). Also liquid biopsy strategies measuring circulating tumor DNA (ctDNA), will provide other lines of opportunities in diagnosis and disease monitoring of DLBCL patients (72–74) Future studies are required to investigate the potential integration of these approaches for the management of DLBCL patients.

### Consensus classification serves the DLBCL patient

The step forward to allow evaluation of new treatment modalities based on DLBCL genetics is now impeded by a discordancy between the 2 independently suggested genomic subtyping approaches, which dictates the challenge that lies ahead of us. Based on various other tumor entities we suggest a blueprint for harmonization of the proposed DNA subtypes, which may allow more widespread clinical implementation. Once this hurdle is taken, a diagnostic work up, applicable in a clinically relevant timeframe, will enable the design of next-generation prospective biomarker-based clinical trials. If successful, the precision medicine with targeted therapies that match dependencies of the molecular subtypes of DLBCL may be brought forward.

## Author contributions

MM, MR, DJ, and BY contributed to conception and design of the review and wrote the first draft of the manuscript. All authors contributed to manuscript revision, read, and approved the submitted version.

## Funding

This work was supported by the Dutch Cancer Society grant KWF 2012-5711.

## Acknowledgments

The authors like to thank dr. Erik van Dijk for critically reading the manuscript prior to submission and Prof. Dr. Pieter Wesseling for helpful discussions on CNS diagnostics, both affiliated to Amsterdam UMC, Vrije Universiteit Amsterdam, Department of Pathology, Amsterdam, The Netherlands.

## Conflict of interest

The authors declare that the research was conducted in the absence of any commercial or financial relationships that could be construed as a potential conflict of interest.

## Publisher’s note

All claims expressed in this article are solely those of the authors and do not necessarily represent those of their affiliated organizations, or those of the publisher, the editors and the reviewers. Any product that may be evaluated in this article, or claim that may be made by its manufacturer, is not guaranteed or endorsed by the publisher.
